# Draft genome and multi-tissue transcriptome assemblies of the Neotropical leaf-frog *Phyllomedusa bahiana*

**DOI:** 10.1093/g3journal/jkac270

**Published:** 2022-10-07

**Authors:** Pedro Andrade, Mariana L Lyra, Juliana Zina, Deivson F O Bastos, Andrés E Brunetti, Délio Baêta, Sandra Afonso, Tuliana O Brunes, Pedro P G Taucce, Miguel Carneiro, Célio F B Haddad, Fernando Sequeira

**Affiliations:** CIBIO, Centro de Investigação em Biodiversidade e Recursos Genéticos, InBIO Laboratório Associado, Campus de Vairão, Universidade do Porto, Vairão 4485-661, Portugal; BIOPOLIS Program in Genomics, Biodiversity and Land Planning, CIBIO, Campus de Vairão, Vairão 4485-661, Portugal; Departamento de Biodiversidade and Centro de Aquicultura, Instituto de Biociências, Universidade Estadual Paulista (UNESP), Rio Claro 13506-900, Brazil; Departamento de Ciências Biológicas, Universidade Estadual do Sudoeste da Bahia, Jequié 45206-190, Brazil; Departamento de Ciências Biológicas, Universidade Estadual do Sudoeste da Bahia, Jequié 45206-190, Brazil; Laboratory of Evolutionary Genetics, Institute of Subtropical Biology, National University of Misiones (UNaM-CONICET) Posadas N3300LQH, Misiones, Argentina; CIBIO, Centro de Investigação em Biodiversidade e Recursos Genéticos, InBIO Laboratório Associado, Campus de Vairão, Universidade do Porto, Vairão 4485-661, Portugal; BIOPOLIS Program in Genomics, Biodiversity and Land Planning, CIBIO, Campus de Vairão, Vairão 4485-661, Portugal; Departamento de Biodiversidade and Centro de Aquicultura, Instituto de Biociências, Universidade Estadual Paulista (UNESP), Rio Claro 13506-900, Brazil; CIBIO, Centro de Investigação em Biodiversidade e Recursos Genéticos, InBIO Laboratório Associado, Campus de Vairão, Universidade do Porto, Vairão 4485-661, Portugal; BIOPOLIS Program in Genomics, Biodiversity and Land Planning, CIBIO, Campus de Vairão, Vairão 4485-661, Portugal; Departamento de Zoologia, Instituto de Biociências, Universidade de São Paulo, São Paulo 05508-090, Brazil; Departamento de Biodiversidade and Centro de Aquicultura, Instituto de Biociências, Universidade Estadual Paulista (UNESP), Rio Claro 13506-900, Brazil; CIBIO, Centro de Investigação em Biodiversidade e Recursos Genéticos, InBIO Laboratório Associado, Campus de Vairão, Universidade do Porto, Vairão 4485-661, Portugal; BIOPOLIS Program in Genomics, Biodiversity and Land Planning, CIBIO, Campus de Vairão, Vairão 4485-661, Portugal; Departamento de Biodiversidade and Centro de Aquicultura, Instituto de Biociências, Universidade Estadual Paulista (UNESP), Rio Claro 13506-900, Brazil; CIBIO, Centro de Investigação em Biodiversidade e Recursos Genéticos, InBIO Laboratório Associado, Campus de Vairão, Universidade do Porto, Vairão 4485-661, Portugal; BIOPOLIS Program in Genomics, Biodiversity and Land Planning, CIBIO, Campus de Vairão, Vairão 4485-661, Portugal

**Keywords:** amphibian, Anura, Phyllomedusidae, transcriptome, genome, mitogenome

## Abstract

Amphibians are increasingly threatened worldwide, but the availability of genomic resources that could be crucial for implementing informed conservation practices lags well behind that for other vertebrate groups. Here, we describe draft de novo genome, mitogenome, and transcriptome assemblies for the Neotropical leaf-frog *Phyllomedusa bahiana* native to the Brazilian Atlantic Forest and Caatinga. We used a combination of PacBio long reads and Illumina sequencing to produce a 4.74-Gbp contig-level genome assembly, which has a contiguity comparable to other recent nonchromosome level assemblies. The assembled mitogenome comprises 16,239 bp and the gene content and arrangement are similar to other Neobratrachia. RNA-sequencing from 8 tissues resulted in a highly complete (86.3%) reference transcriptome. We further use whole-genome resequencing data from *P. bahiana* and from its sister species *Phyllomedusa burmeisteri*, to demonstrate how our assembly can be used as a backbone for population genomics studies within the *P. burmeisteri* species group. Our assemblies thus represent important additions to the catalog of genomic resources available from amphibians.

## Introduction

Amphibians are a highly species-rich group of vertebrates, and a large number of its species are affected by widespread, human-driven environmental changes that have impacted global biodiversity. According to the IUCN’s Red List, 41% out of 7,215 species assessed are thought to be endangered (IUCN 2021), and for a further ∼2,200 species for which scarce data prevents a proper IUCN assessment, about half are estimated to be endangered as well (González-del-Pliego *et al.* 2019). Major threats include habitat loss, the spread of the fungal pathogen *Batrachochytrium dendrobatidis*, pesticides and climate change (Collins and Storfer 2003; Bishop *et al.* 2012; Lips 2016). While these threats are impacting amphibian diversity worldwide, effects seem to be particularly severe for Neotropical species (Stuart *et al.* 2004).

In this context, there is thus ample need to ramp up conservation efforts of amphibian groups worldwide and generate resources for researchers working on this taxonomic group. Genomic resources, in particular, can provide unique insights into the evolutionary history of species and the functional basis underlying adaptation to environmental stressors (Supple and Shapiro 2018). Apart from conservation concerns, generating genomic resources for amphibians is also highly valuable to increase our understanding of genome evolution in vertebrates. Amphibians are noteworthy for presenting highly variable genome sizes, which are mostly (but not only) a consequence of the evolution of their transposable element content (Liedtke *et al.* 2018; Herrick and Sclavi 2020). Despite these reasons, amphibians lag behind other major vertebrate taxa when considering the availability of genomic resources. For example, a quick search in NCBI’s Assembly database (https://www.ncbi.nlm.nih.gov/assembly; last accessed 2022 Jul 27) currently recovers 42 published amphibian assemblies, compared with more than 700 birds, 2,300 mammals, 1,300 teleosts, and 90 nonavian sauropsids.

Treefrogs (Anura, Arboranae) is a widespread group of amphibians across the whole American continent, Eurasia, North Africa, and the Australo-Papuan region, with a much higher number of species around its center of diversification in South America, where they are estimated to have originated in the Palaeocene (Duellman *et al.* 2016). They comprise 3 families (Hylidae, Pelodryadidae, and Phyllomedusidae), with estimated 1,035 species (Frost 2022), making treefrogs one of the more species-rich groups within the Anura. In spite of this, very few genomic resources have been generated for this group.

**Fig. 1.**
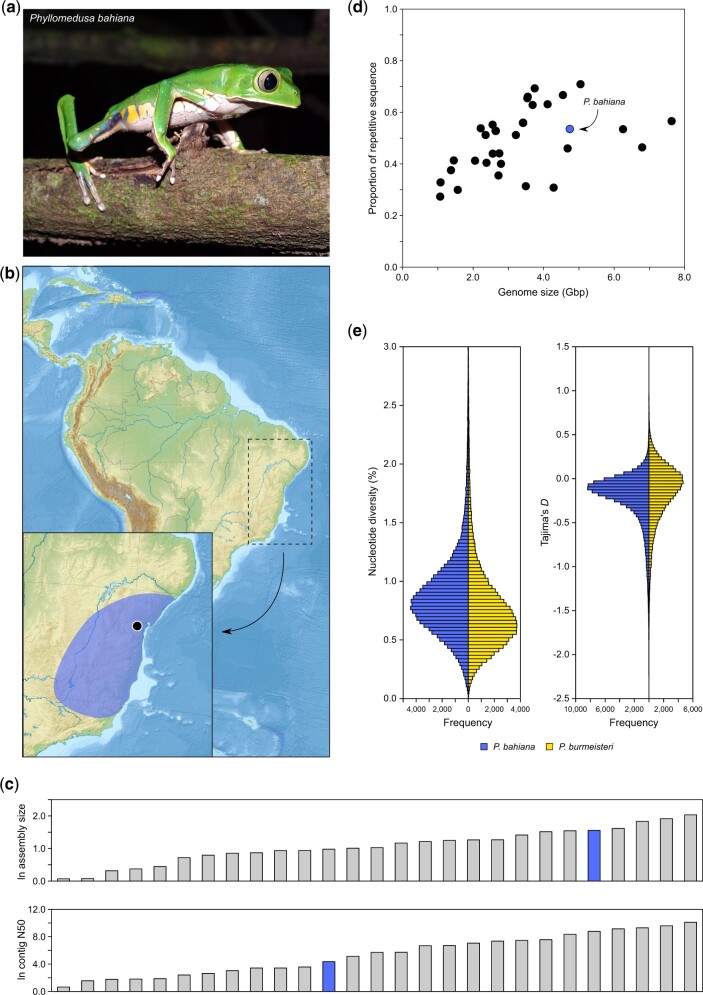
a) Photo of *Phyllomedusa bahiana* (by Pedro P. G. Taucce). b) Map with the approximate distribution range of *P. bahiana* (blue shading), with indication on sampling location for the specimens used for reference genome and transcriptome assemblies (Jequié, Bahia, Brazil). Distribution map was adapted from Pombal and Haddad (1992), while the topographic map was retrieved from Natural Earth (https://www.naturalearthdata.com). c) Comparison between ours and other anuran assemblies regarding assembly size and contig N50 (natural logarithm transformed for ease of visualization). The position of *P. bahiana* is highlighted in blue (see Supplementary Figs. 1 and 2 for names of all species). d) Relationship between genome size (Gbp) and the proportion of repetitive sequence for all anuran species with published genomes. Repetitive sequence content was calculated for each genome using a de novo, unbiased repeat detection method. e) Frequency distribution of genome-wide values of nucleotide diversity (π) and Tajima’s *D* for *P. bahiana* and *P. burmeisteri*, based on pool-sequencing data. Each statistic was calculated in 10-kb nonoverlapping windows across the whole genome. For better visualization, a small number of windows with nucleotide diversity values above 3.0% was omitted.

In this study, we present draft genome, mitogenome, and transcriptome assemblies of the Neotropical leaf-frog *Phyllomedusa bahiana* Lutz, 1925 (Anura, Phyllomedusidae; Fig. 1a). This species is part of the *P. burmeisteri* species group (Faivovich *et al.* 2010), which includes 4 diploid and one tetraploid species. This group is essentially distributed along the *Mata Atlântica* (Brazilian Atlantic Forest), *Caatinga* (Brazilian dry forest), and *Pampa* (grassy plains) biomes of South America, mostly in Brazil, and are estimated to have diverged ca. 4.9 MYA (Fig. 1b;Brunes *et al.* 2010). Cytogenetic analyses indicate a conserved karyotype of 2*n* = 26 for diploid species of this group (Gruber *et al.* 2013; Barth *et al.* 2013, 2014), and a haploid genome size between 6.0 and 7.0 Gbp (Goin *et al.* 1968; Batistic *et al.* 1975; Liedtke *et al.* 2018). Leaf frogs have a combination of traits that make them promising models to study a variety of biological processes. For example, they have a very high capacity to deal with temperature stress by reducing evaporative water loss considerably when compared with other anurans, a skill that is achieved through secreting lipids that are spread over the skin coupled with increased water retention by excreting uric acid instead of urea (Withers *et al.* 1984; Shoemaker *et al.* 1972). Another important aspect of the biology of leaf frogs is the abundant and complex mixture of more than 200 bioactive peptides that evolved for defense against predators and pathogens, and that are of interest in the pharmaceutical industry (Calderon *et al.* 2011; Bartels *et al.* 2019; Zandsalimi *et al.* 2020). These aspects underscore the opportunities that may arise from a more complete characterization of the molecular diversity in these amphibians at multiple levels, for which the establishment of genomic resources has the potential to be a decisive first step.

## Materials and methods

### Whole-genome long- and short-read sequencing

For the genome assembly, an adult male of *P. bahiana* was collected from the municipality of Jequié (Bahia, Brazil, −13.987925S −40.007450W; Fig. 1b). The individual was euthanized by applying 5% lidocaine to the skin. Heart tissue was collected and immediately frozen in −80°C. The specimen was fixed in 10% formalin and transferred to 70% ethanol for permanent storage at Museu de História Natural de Jequié Coleção Herpetológica, Universidade Estadual do Sudoeste da BahiaM (Voucher: MHNJCH 1462).

High molecular weight DNA (fragment size >60 kb) was extracted from heart tissue using the MagAttract HMW DNA Kit (QIAGEN). DNA quantity and integrity was assessed using a NanoDrop instrument, Qubit dsDNA BR Assay Kit and Genomic DNA ScreenTape (Agilent). To assemble the genome of *P. bahiana*, we used long-read sequencing as the main source of data. PacBio libraries were prepared with the SMRTbell Express Template Prep Kit 2.0 and sequenced on a Sequel II instrument on 2 independent runs, each with a movie time of 30 h to generate CCS reads (performed at Novogene UK). Additionally, we also generated a 150-bp short-read library with a 10× Genomics Chromium linked-read protocol (Novogene UK), which was sequenced to an estimated depth of 53.7× with an Illumina NovaSeq instrument (assuming a genome size of 6.5 Gbp).

### Genome assembly

To assemble our genome, we input the raw PacBio sub-read data, converted into fastq format using the software *bamUtil* v1.0.14 (https://github.com/statgen/bamUtil) into *wtdbg2* v2.5 (Ruan and Li 2020) assuming a genome size of 6.5 Gb and maintaining other parameters as default. An additional assembly was performed by reducing the minimum length of alignment (−l, from 2,048 to 1,000), the minimum subread length (−L, from 5,000 to 4,000) and minimum depth (−X, from 50 to 30); however, results were qualitatively unchanged, so we retained the assembly that resulted from the run with default parameters. In parallel, we also attempted to generate a Chromium assembly with the linked-read data using *Supernova* v2.0 (Weisenfeld *et al.* 2017), but assembly statistics (e.g. scaffold N50, contig N50) were not satisfactory, so we discarded this assembly.

The sequence accuracy of assemblies based on long reads are typically error-prone, so following assembly we corrected the sequence using the Illumina Chromium whole-genome sequencing data. For polishing, we used *NextPolish* v1.3.1 (Hu *et al.* 2020) imposing a maximum depth of 100 and a single iteration of polishing (contrary to other tools, *NextPolish* does not require repeated polishing iterations). Due to the general lack of knowledge on amphibian genome evolution patterns from the lack of available reference genome sequences, repeat element detection and masking was done using a de novo method based on the detection of repeated *k*-mers, using the software *Red* v2.0 (Girgis 2015) using default parameters. This approach, while not identifying specific classes of repeat elements, is unconstrained by the comparison to a reference repeat library, which makes it likely more accurate at detecting true repeats. To perform a comparative analysis of repeat element abundance in anurans, we also carried out a *k*-mer-based repeat detection step using *Red* for other anuran genome assemblies retrieved from the NCBI assembly database and other sources (Table 1).

**Table 1.** Summary of the sequencing datasets of *Phyllomedusa bahiana* and *P. burmeisteri* used in this study, including accession codes for NCBI’s Sequence Read Archive (SRA).

Dataset	Accession codes	Data type	Sequencing instrument	Number of reads	Tissue type
P.bahiana long-read sequencing (libraryA)	SRR18363239	PacBio CCS	Sequel II	18,011,507	Heart
P.bahiana long-read sequencing (libraryB)	SRR18363238	PacBio CCS	Sequel II	10,641,819	Heart
P.bahiana Chromium sequencing	SRR18363237	Chromium linked reads (150-bp PE)	Illumina NovaSeq	2,330,800,682	Heart
P.bahiana RNAseq (liver)	SRR18363236	Illumina RNAseq (100-bp PE)	Illumina NovaSeq	65,108,950	Liver
P.bahiana RNAseq (heart)	SRR18363235	Illumina RNAseq (100-bp PE)	Illumina NovaSeq	71,641,510	Heart
P.bahiana RNAseq (testicle)	SRR18363234	Illumina RNAseq (100-bp PE)	Illumina NovaSeq	65,952,858	Testicle
P.bahiana RNAseq (spleen)	SRR18363233	Illumina RNAseq (100-bp PE)	Illumina NovaSeq	62,894,824	Spleen
P.bahiana RNAseq (kidney)	SRR18363232	Illumina RNAseq (100-bp PE)	Illumina NovaSeq	60,424,896	Kidney
P.bahiana RNAseq (skin-dorsum)	SRR18363229	Illumina RNAseq (100-bp PE)	Illumina NovaSeq	55,109,630	Skin (dorsum)
P.bahiana RNAseq (skin-thigh)	SRR18363231	Illumina RNAseq (100-bp PE)	Illumina NovaSeq	67,226,174	Skin (inner thigh)
P.bahiana RNAseq (skin-trunk)	SRR18363230	Illumina RNAseq (100-bp PE)	Illumina NovaSeq	61,183,514	Skin (lateral trunk)
P.bahiana WGS pool-seq	SRR18363241	Illumina WG pool-seq (150-bp PE)	Illumina NovaSeq	742,662,620	Muscle
P.burmeisteri WGS pool-seq	SRR18363240	Illumina WG pool-seq (150-bp PE)	Illumina NovaSeq	846,586,334	Muscle

After an initial submission to NCBI’s GenBank database, an automated screening for adaptor sequences detected potential contamination (adaptor sequences NGB00972.1 and NGB01063) in 44 genomic regions (from 40 contigs). To correct this, we converted these adaptor sequences into Ns and used these to split the contigs using the script *split.scaffolds.to.contigs.pl* (Mads Albertsen, https://github.com/MadsAlbertsen).

To evaluate the quality of our reference assembly, we calculated common metrics using the script assemblathon_stats.pl (https://github.com/KorfLab/Assemblathon; Bradnam *et al.* 2013). To assess genome completeness, we also quantified the number of highly conserved single-copy orthologs using *BUSCO* v5 (Simão *et al.* 2015). Gene prediction for this orthology search was performed using *MetaEuk* v4-a0f584d (Levy Karin *et al.* 2020), with the lineage dataset metazoa_odb10.

### Mitochondrial genome sequencing and assembly

To assembly the mitochondrial genome (mitogenome) we used the forward read sequences from the Illumina sequencing data. The raw reads were trimmed for adapters using *Trimmomatic* v0.39 (Bolger *et al.* 2014) and all reads smaller than 50 bp were discarded. The mitogenome was assembled in 2 steps: we first used *GetOrganelle* v1.7.5 (Jin *et al.* 2020) under default settings to produce a draft of the *P. bahiana* mitogenome and then we used this draft as seed for an iterative mapping assembly using *MITObim* v1.9.1 (Hahn *et al.* 2013). Iterations were run until no additional reads could be incorporated into the assembly. We evaluated the assemblies for completeness and coverage by importing the mapping output from *MITObim* in *Geneious* vR11 (https://www.geneious.com). The final mitogenome annotation was carried out using *MITOS* v2 (Donath *et al.* 2019). The protein-coding regions were checked to confirm that no indels or stop codons were present.

### RNA-sequencing

For the transcriptome assembly, we collected a male *P. bahiana*, euthanized it as previously described and harvested the following tissues: liver, heart, testicle, spleen, kidney, and skin (from the latter, 3 individual patches from the dorsum, inner thigh, and lateral trunk). All tissues were immediately placed in −80°C. Total RNA was isolated using the RNeasy Mini kit (QIAGEN) followed by DNAse digestion. RNA purity and concentration were assessed using a Nanodrop instrument prior to library preparation. cDNA was generated from ∼1 μg of RNA, and strand-specific Illumina libraries were prepared using the TruSeq RNA Library Prep Kit v2. Libraries were sequenced using 100 bp paired-end reads on an Illumina NovaSeq instrument (Macrogen, Inc., Seoul, Republic of Korea).

### Transcriptome assembly

RNAseq read quality was confirmed using *FastQC* v0.11.8. Since we intended to perform a general transcriptome assembly, for the following steps we merged reads from all tissues. Merged reads were corrected with *Rcorrector* v1.0.3.1 (Song and Florea 2015) to exclude read pairs with at least one unfixable read, and *Trim Galore!* v0.6.0 (https://www.bioinformatics.babraham.ac.uk/projects/trim_galore) to remove adapters, low quality bases (Phred score <5), and reads smaller than 36 bp after trimming. To remove contamination from ribosomal RNA, reads were mapped with *Bowtie2* v2.3.5 (Langmead and Salzberg 2012) to the SSUParc and LSUParc fasta files from the database SILVA (Quast *et al.* 2013) (https://www.arb-silva.de/; downloaded April 2019) using the very-sensitive-local option. Reads that were positive hits were discarded. For assembly, we used *SPAdes* v3.15.2 (Prjibelski *et al.* 2020) with kmer sizes of 21, 33, 51, 77, 99, and 127. To reduce redundancy in the assembly, we clustered highly similar sequences (0.99% identity threshold) with *CD-HIT* v4.8.1 (Fu *et al.* 2012).

We assessed completeness of the transcriptome assembly by comparing it to the metazoa_odb10 database with *BUSCO*, as performed for the genome assembly. To generate annotations for our transcripts, we used S*ma3s* v2 (Casimiro-Soriguer *et al.* 2017), using *BLAST* v2.2.29 (Camacho *et al.* 2009) to search (low complexity filter activated) against the whole UniProt database to retrieve gene names and GO (gene ontology) terms. To infer orthology and duplication events, we compared our de novo transcriptome with a set of published anuran transcriptomes (see Supplementary Table 2 for a list of species and accession codes for the datasets) using *OrthoFinder* v2.5.4 (Emms and Kelly 2019), employing *FastTree* (Price *et al.* 2010) for tree inference.

### Whole-genome pool-sequencing

To investigate the potential of our reference assembly to conduct population genomics studies, we carried out whole-genome sequencing based on pools of individuals. To do so we collected samples of *P. bahiana* from the Brazilian locality of Jequié, Bahia (*n* = 23) and allopatric populations from its sister species *P. burmeisteri* from different Brazilian municipalities (*n* = 18; Campinas, Queluz, São José do Rio Pardo, Nazaré Paulista, Atibaia and Patrocínio Paulista—all in São Paulo state). From each sample, we extracted DNA using the QIAamp DNA Micro Kit (QIAGEN). DNA concentration was quantified using a Qubit dsDNA BR Assay Kit (ThermoFisher), DNA purity was assessed using NanoDrop spectrophotometry (ThermoFisher) and average fragment size assessed using agarose gel electrophoresis. After these assessments, we pooled DNA from each individual in equimolar concentrations. Illumina TruSeq PCR-free libraries from the 2 pools were prepared according to the manufacturer’s protocol, quantified by qPCR using the KAPA Library Quantification Kit (Roche) and sequenced using 2 × 150 bp reads in an Illumina NovaSeq instrument (Novogene UK).

### Population genomics

We evaluated the quality of the sequencing runs using *FastQC* v0.11.8. We mapped the reads to the de novo reference assembly with *BWA-MEM* v1 (Li 2013) using default settings and calculated mapping statistics using *SAMtools* v1.9 (Li *et al.* 2009) and custom scripts. Pool-sequencing does not allow to extract individual genotypes, so we calculated statistics based on allele frequencies. *SAMtools mpileup* was used to gather per-site allele counts, from which allele frequencies were calculated and population genetics parameters derived using *PoPoolation* v1.2.2 (Kofler, Orozco-terWengel, *et al.* 2011) and *PoPoolation2* v1.201 (Kofler, Pandey, *et al.* 2011). Nucleotide diversity (π; Nei 1987), Tajima’s *D* (Tajima 1989), and *F*_ST_ (Hartl and Clark 2007) were calculated in nonoverlapping 10 kb windows, imposing a minimum coverage of 10, a maximum coverage of 100, a minimum base quality of 30, a minimum count of 2 for the alternate allele to consider a position as variable, and excluding windows with a minimum covered fraction below 0.2.

## Results and discussion

### Genome and mitogenome assemblies

Our two PacBio runs generated a total of 28,653,326 subreads, with an average subread length of 14,046.7 bp (Table 1). Chromium Illumina sequencing of the same individual, which was used for polishing the assembly, yielded 2,330,800,682 reads (Table 1). After assembly and polishing, we generated an unscaffolded reference of 4.74 Gbp derived from 109,372 contigs. Contig N50 length was 78.5 kb. The assembled genome size was lower than cytogenetic estimates for *P. burmeisteri* and *P. bicolor* (6.5–7.0 Gbp; Goin *et al.* 1968; Batistic *et al.* 1975; Liedtke *et al.* 2018). Comparing to 27 other published anuran assemblies, the *P. bahiana* assembly sits at the median of the distribution when looking both at contig N50 length (16th largest out of 27; Table 2 and Fig. 1c) and the percentage of the genome in scaffolds higher than 100 kb (15th largest out of 27; Table 2). Differences to genomes with higher values of these statistics are mostly explained by additional sources of sequencing data (typically HiC), higher PacBio sequencing depth, or both.

**Table 2.** Summary statistics for our de novo genome assembly for *Phyllomedusa bahiana*, with data from other anuran genome assemblies for comparison (data retrieved from NCBI GenBank, the Vertebrates Genome Project and the China National GeneBank).

Species	Clade	Accession no.	Assembly size (Gb)	Scaffold N50 (Mb)	Number of contigs	Contig N50 (kb)	% contigs >100 kb
*Phyllomedusa bahiana*	Phyllomedusidae	JAODAL000000000	4.74	–	109329.0	78.5	10.5
*Bombina variegate*	Bombinatoridae	GCA_905336975.1	4.68	0.0	4,302,271	1.9	0.0
*Bufo bufo*	Bufonidae	GCA_905171765.1	5.04	635.7	5,402	4,245.5	50.5
*Bufo gargarizans*	Bufonidae	GCA_014858855.1	4.55	539.8	4,619	1,743.3	90.8
*Rhinella marina*	Bufonidae	GCA_900303285.1	2.55	0.2	31,391	167.5	18.6
*Oophaga pumilio*	Dendrobatidae	GCA_009801035.1	3.49	0.1	629,903	5.9	0.0
*Ranitomeya imitator*	Dendrobatidae	GCA_905332335.1	6.79	0.4	77,709	300.0	15.9
*Nanorana parkeri*	Dicroglossidae	GCA_000935625.1	2.05	1.1	138,648	35.6	0.9
*Eleutherodactylus coqui*	Eleutherodactylidae	GCA_019857665.1	2.79	109.5	395,443	13.9	0.1
*Dendropsophus ebraccatus*	Hylidae	aDenEbr1.mat	2.35	61.6	3,268	10,846.3	27.1
*Dendropsophus ebraccatus*	Hylidae	aDenEbr1.pat	2.21	153.4	2,934	9,352.4	29.7
*Engystomops pustulosus*	Leptodactylidae	GCA_019512145.1	2.56	172.1	125,382	308.9	4.4
*Limnodynastes dumerilii*	Limnodynastidae	GCA_011038615.1	2.38	0.3	730,467	11.0	0.0
*Platyplectrum ornatum*	Limnodynastidae	GCA_016617825.1	1.07	0.0	238,193	4.7	0.0
*Leptobrachium ailaonicum*	Megophryidae	GCA_018994145.1	3.54	412.4	15,899	821.1	35.9
*Leptobrachium leishanense*	Megophryidae	GCA_009667805.1	3.55	394.7	8,584	1,946.3	42.6
*Spea multiplicate*	Pelobatidae	GCA_009364415.1	1.08	0.1	74,867	30.7	1.5
*Hymenochirus boettgeri*	Pipidae	GCA_019447015.1	3.21	293.3	42,109	801.2	11.6
*Pipa parva*	Pipidae	GCA_019650415.1	1.37	0.0	337,090	6.4	0.0
*Xenopus laevis*	Pipidae	GCA_017654675.1	2.74	155.3	631	24,555.0	55.3
*Xenopus tropicalis*	Pipidae	GCA_000004195.4	1.45	154.0	845	14,634.3	49.0
*Pyxicephalus adspersus*	Pyxicephalidae	GCA_004786255.1	1.56	157.5	116,216	30.8	0.8
*Glandirana rugosa*	Ranidae	GCA_018402905.1	7.63	0.7	987,724	20.7	0.1
*Rana catesbeiana*	Ranidae	GCA_002284835.2	6.25	0.0	2,026,780	6.1	0.0
*Rana temporaria*	Ranidae	GCA_905171775.1	4.11	481.8	2,334	6,519.6	70.9
*Rhacophorus dugritei*	Rhacophoridae	CNA0045871	3.36	2.4	25,223	1,565.8	16.6
*Rhacophorus kio*	Rhacophoridae	CNA0045870	2.66	300.1	8,366	1,168.0	45.7

A reference list for these genome assemblies can be found in Supplementary Table 1.

*BUSCO* analysis revealed that our genome was reasonably complete. A total of 954 single-copy orthologs from a metazoan database were searched against our genome, from which 671 (70.3%) were complete and single-copy. In spite of the large genome size of *P. bahiana* only 15 of the reference single-copy orthologs (1.6%) were duplicated. Fragmented orthologs account for a large portion of noncomplete *BUSCO* groups (154, or 16.1% of the total number), which is a likely consequence of a fragmented assembly. When comparing to other amphibian genome assemblies for which similar completeness analyses were performed, completeness values of our assembly are in line with those of other assemblies with reduced or no scaffolding (e.g. Hammond *et al.* 2017; Lamichhaney *et al.* 2021), which represents ∼10–20% lower completeness than of highly scaffolded assemblies (e.g. Li, Ren, *et al.* 2019; Seidl *et al.* 2019; Li *et al.* 2020; Streicher *et al.* 2021a, 2021b; Wu *et al.* 2022).

To further test if the large genome size of *P. bahiana* is explained by either widespread duplications or expansions of repetitive genomic elements, we calculated the percentage of the total genome that is repetitive in our assembly and the other anuran genomes. As expected, the percentage of the genome that was found to be repetitive increased linearly and significantly with assembled genome size across all species (ordinary least squares regression, *r*^2^ = 0.23, *P = *0.008), with *P. bahiana* having the fifth largest assembly size and twelfth largest proportion of the genome that is repetitive (Fig. 1d; P*. bahiana *=* *0.53; min. = 0.27; max = 0.71). Considering these findings together with *BUSCO* results indicating low amounts of gene duplication, the expansion of repetitive elements is one likely contributing factor for the large genome size of *P. bahiana*, as has been suggested for other amphibian species (Metcalfe and Casane 2013; Rogers *et al.* 2018; Li, Yu, *et al.* 2019). It should, however, be taken into account that a more detailed characterization of the repeat element landscape of the genome is needed to draw definitive conclusions. If this is confirmed, it could explain the discrepancy between estimated genome sizes from flow cytometry and the final assembly size in other large anuran genomes (Edwards *et al.* 2018; Rogers *et al.* 2018; Streicher *et al.* 2021a, since the assembly of repetitive sequences is highly complex (Tørresen *et al.* 2019).

The complete mitogenome assembly resulted in a total of 16,239 bp with mean coverage of 164×, containing the expected 2 ribosomal genes (12S and 16S rRNA), the 13 protein-coding genes, 22 tRNAs and the noncoding control region. The gene order arrangement and gene content are consistent with *Phyllomedusa tomopterna* and *Pithecopus megacephalus*, so far the only mitogenomes available for the Phyllomedusidae; they also agree with the pattern found for other Neobatrachia (Zhang *et al.* 2013; Lima *et al.* 2020).

### Transcriptome assembly and orthology inference

RNA-sequencing from multiple tissues generated a total of 509,542,356 raw reads (number of reads per library ranging between 55,109,630 and 71,641,510; Table 1). The assembly of an overall transcriptome from the merged dataset yielded a total of 168,692 transcripts, with a mean transcript length of 657 bp (longest transcript = 11,718 bp) and 43.47% GC content. The *BUSCO* search for single-copy orthologs indicated that 86.3% of the searched groups were complete and single-copy. Similarly to the genome assembly, we found little evidence of widespread genome duplication, since only 5 *BUSCO* groups (0.5% of the total) were duplicated. Of the total number of transcripts, we were able to annotate 35,598 transcripts (21.10%) with either putative orthologs from the Uniprot database, GO term annotations or both (the majority of cases). Comparison of orthologous transcripts between our assembled transcriptome and those of other anurans recovered a total of 2,746,416 independent transcripts, the majority of which (73.7%) were assigned to orthogroups. For *P. bahiana*, 75.5% of transcripts were placed in orthogroups, with 24.6% in species-specific orthogroups.

### Population genomics

To estimate population genetics parameters from samples of *P. bahiana* and its sister species *P. burmeisteri*, we performed whole-genome sequencing of 2 pools of individuals, 1 pool from each species. We generated a total of 742,662,620 reads for the *P. bahiana* pool (∼23.50× coverage) and 846,586,334 reads for *P. burmeisteri* (∼26.78× coverage), which after read trimming, were mapped to the reference genome assembly with high mapping rates for both pools (93.98% and 95.78%, respectively). We observed in both cases a substantial drop in the percentage of properly paired reads (74.08% and 72.81%), which likely reflects the fragmented nature of our current assembly.

Using this data, we next compared patterns of sequence variation in our 2 samples. Variant calling based on allele counts retrieved a total of 42,502,110 biallelic single nucleotide polymorphisms. Out of these, 6,388,773 (15.0%) are diagnostic mutations between the species (allele frequency difference = 1.0). Genome-wide *F*_ST_ between the 2 species was high (average ± SD; *F*_ST_ = 0.45 ± 0.15). We did not find evidence that genetic differentiation was clustered in any particular region of the genome (Supplementary Fig. 3). For example, when looking at counts of diagnostic mutations in nonoverlapping 10 kb windows for the largest 250 contigs, the windows with higher counts of diagnostic alleles were scattered across these contigs (and apart from these contigs, only 12 additional genomic windows had higher values, and none located in the same contig). A similar pattern was found for *F*_ST_, which is consistent with these populations representing lineages with largely independent evolutionary histories. However, despite a deep divergence time between the taxa (∼2.9 MY; Brunes *et al.* 2014), average genome-wide nucleotide diversity is similar between them (average ± SD; π_bahiana_ = 0.89 ± 0.50; π_burmeisteri_ = 0.82 ± 0.56; Fig. 1e and Supplementary Fig. 4). These values are relatively high when compared with most vertebrates (Romiguier *et al.* 2014). Tajima’s *D* was also similar (slightly negative) between the 2 species (average ± SD; TD_bahiana_ = −0.26 ± 0.40; TD_burmeisteri_ = −0.21 ± 0.49; Fig. 1e and Supplementary Fig. 5). These results are consistent with previous findings using nuclear markers (Brunes *et al.*, 2014), and are suggestive of population expansion following a bottleneck, a pattern commonly found among many other amphibians from the Atlantic Forest (e.g. Carnaval *et al.* 2009; Brunes *et al.* 2015).

Arboranae represent around 10% of the total number of known amphibian species. Our de novo reference assemblies of the nuclear genome, mitogenome, and transcriptome of *P. bahiana*, which are among the first genomic data for the Arboranae, constitute important resources that can serve as the basis for future studies on the ecology and evolution of Neotropical leaf frogs. Future enhancements to our assemblies should focus on improving genome scaffolding and contiguity in order to achieve a chromosome-scale assembly. This should be achieved through the use of additional sources of data, such as chromosome conformation capture (HiC) and also by increasing the sequencing depth with long-reads (either PacBio or Oxford Nanopore) (Luo *et al.* 2021).

## Supplementary Material

jkac270_Supplementary_Data

## Data Availability

Raw whole-genome sequencing data (PacBio long reads, Chromium linked reads and Illumina pool-sequencing) and RNA-sequencing data have been deposited in the Sequence Read Archive (www.ncbi.nlm.nih.gov/sra) under BioProject PRJNA771272. Fasta files for the assemblies have been deposited in GenBank, with the following accession codes: genome (JAODAL000000000), mitogenome (OM460708), and transcriptome (GJVR00000000). Supplemental material is available at G3 online.
