# Supplementary material for: Draft genome and multi-tissue transcriptome assemblies of the Neotropical leaf-frog Phyllomedusa bahiana
Source: G3 (Bethesda). 2022 Oct 7;12(12):jkac270. doi: 10.1093/g3journal/jkac270 (PMC9713437; doi:10.1093/g3journal/jkac270)
Supplement: jkac270_Supplementary_Data [file jkac270_supplementary_data.doc]

**SUPPLEMENTARY DATA**

G3: Genes | Genomes | Genetics

**Draft genome and multi-tissue transcriptome assemblies of the Neotropical leaf-frog *Phyllomedusa bahiana***

Pedro Andrade^*^, Mariana L. Lyra, Juliana Zina, Deivson F. O. Bastos, Andrés E. Brunetti, Délio Baêta, Sandra Afonso, Tuliana O. Brunes, Pedro P. G. Taucce, Miguel Carneiro, Célio F. B. Haddad, Fernando Sequeira^*^

*corresponding authors: PA (pandrade@cibio.up.pt) and FS (fsequeira@cibio.up.pt)

**Supplementary tables**

**Table S1**. Reference list for anuran genome assemblies that were used for comparison with the de novo *Phyllomedusa bahiana* assembly generated in this study (data retrieved from NCBI GenBank, the Vertebrates Genome Project and the China National GeneBank).

| **Species** | **Clade** | **Accession no.** | **Reference** |
| --- | --- | --- | --- |
| *Phyllomedusa bahiana* | Phyllomedusidae | JAODAL000000000 | *This study* |
| *Bombina variegata* | Bombinatoridae | GCA_905336975.1 | Nürnberger et al., 2021 |
| *Bufo bufo* | Bufonidae | GCA_905171765.1 | Streicher et al., 2021a |
| *Bufo gargarizans* | Bufonidae | GCA_014858855.1 | Lu et al., 2021 |
| *Rhinella marina* | Bufonidae | GCA_900303285.1 | Edwards et al., 2018 |
| *Oophaga pumilio* | Dendrobatidae | GCA_009801035.1 | Rogers et al., 2018 |
| *Ranitomeya imitator* | Dendrobatidae | GCA_905332335.1 | Stuckert et al., 2021 |
| *Nanorana parkeri* | Dicroglossidae | GCA_000935625.1 | Sun et al., 2015 |
| *Eleutherodactylus coqui* | Eleutherodactylidae | GCA_019857665.1 | Bredeson et al., 2021 |
| *Dendropsophus ebraccatus* | Hylidae | aDenEbr1.mat | Vertebrate Genomes Project (2022) |
| *Dendropsophus ebraccatus* | Hylidae | aDenEbr1.pat | Vertebrate Genomes Project (2022) |
| *Engystomops pustulosus* | Leptodactylidae | GCA_019512145.1 | Bredeson et al., 2021 |
| *Limnodynastes dumerilii* | Limnodynastidae | GCA_011038615.1 | Li et al., 2020 |
| *Platyplectrum ornatum* | Limnodynastidae | GCA_016617825.1 | Lamichhaney et al., 2021 |
| *Leptobrachium ailaonicum* | Megophryidae | GCA_018994145.1 | Li et al., 2019a |
| *Leptobrachium leishanense* | Megophryidae | GCA_009667805.1 | Li et al., 2019b |
| *Spea multiplicata* | Pelobatidae | GCA_009364415.1 | Seidl et al., 2019 |
| *Hymenochirus boettgeri* | Pipidae | GCA_019447015.1 | Bredeson et al., 2021 |
| *Pipa parva* | Pipidae | GCA_019650415.1 | Mudd, 2019 |
| *Xenopus laevis* | Pipidae | GCA_017654675.1 | Session et al., 2016 |
| *Xenopus tropicalis* | Pipidae | GCA_000004195.4 | Bredeson et al., 2021 |
| *Pyxicephalus adspersus* | Pyxicephalidae | GCA_004786255.1 | Denton et al., 2018 |
| *Glandirana rugosa* | Ranidae | GCA_018402905.1 | Katsura et al., 2021 |
| *Rana catesbeiana* | Ranidae | GCA_002284835.2 | Hammond et al., 2017 |
| *Rana temporaria* | Ranidae | GCA_905171775.1 | Streicher et al., 2021b |
| *Rhacophorus dugritei* | Rhacophoridae | CNA0045871 | Wu et al., 2022 |
| *Rhacophorus kio* | Rhacophoridae | CNA0045870 | Wu et al., 2022 |

**Table S2**. Transcriptomic data from multiple anuran species that was used as input for the orthology analysis with *OrthoFinder*.

| **Species** | **Clade** | **Accession no. (NCBI (TSA)** | **Reference** |
| --- | --- | --- | --- |
| *Bufotes viridis* | Bufonidae | GDRL00000000 | Gerchen et al., 2016 |
| *Rhinella arenarum* | Bufonidae | GHCG00000000 | Ceschin et al., 2020 |
| *Oreobates cruralis* | Craugastoridae | GFNJ00000000 | Montero-Mendieta et al., 2017 |
| *Oophaga pumilio* | Dendrobatidae | GIKS00000000 | Rodríguez et al., 2020 |
| *Boana pugnax* | Hylidae | GISC00000000 | Liscano Martinez et al., 2020 |
| *Dryophytes cinereus* | Hylidae | GENE00000000 | Unpublished (Sinkiewicz DM, WilczynskiW) |
| *Pseudacris regilla* | Hylidae | GAEI00000000 | Robertson and Cornman, 2014 |
| *Leptobrachium boringii* | Megophryidae | GEGK00000000 | Huang et al. 2016 |
| *Megophrys sangzhiensis* | Megophryidae | GEGL00000000 | Huang et al. 2016 |
| *Pelobates cultripes* | Pelobatidae | GHBH00000000 | Liedtke et al., 2019 |
| *Phyllomedusa bahiana* | Phyllomedusidae | GJVR00000000 | *This study* |
| *Xenopus tropicalis* | Pipidae | GIVH00000000 | Furman et al., 2020 |
| *Odorrana margaretae* | Ranidae | GEGJ00000000 | Huang et al. 2016 |
| *Pelophylax nigromaculatus* | Ranidae | GEGI00000000 | Huang et al. 2016 |
| *Rana temporaria* | Ranidae | GGNS00000000 | Ma et al., 2018 |
| *Polypedates megacephalus* | Rhacophoridae | GEGH00000000 | Huang et al. 2016 |
| *Rhacophorus dennysi* | Rhacophoridae | GEGG00000000 | Huang et al. 2016 |
| *Zhangixalus omeimontis* | Rhacophoridae | GEGF00000000 | Huang et al. 2016 |
| *Scaphiopus couchii* | Scaphiopodidae | GHBO00000000 | Liedtke et al., 2019 |

**Supplementary figures**


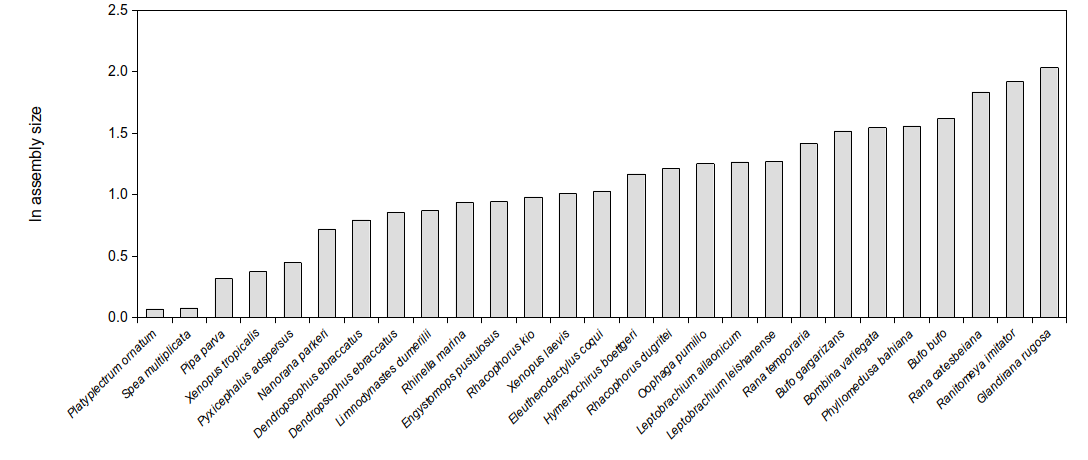
**Fig. S1.** Comparison between several anuran assemblies for assembly size (natural logarithm transformed for ease of visualization).


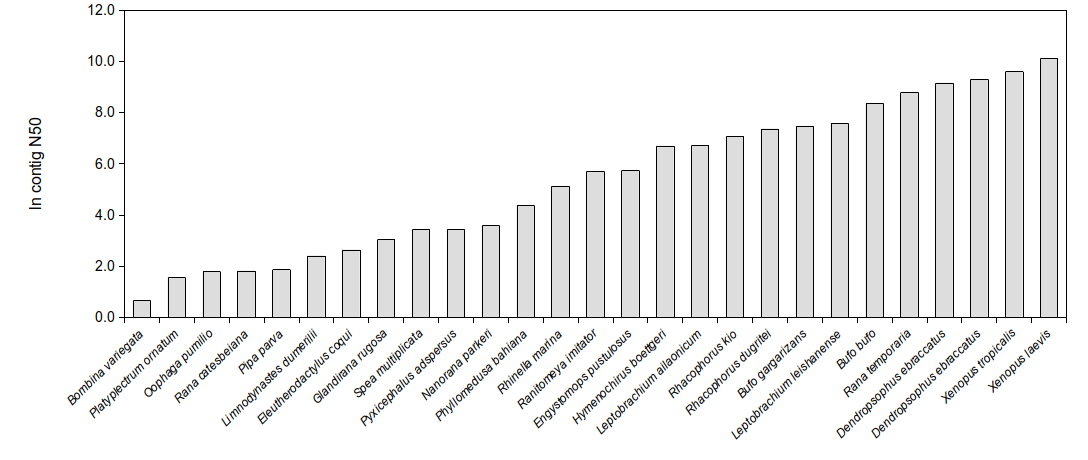
**Fig. S2.** Comparison between several anuran assemblies for contig N50 length (natural logarithm transformed for ease of visualization).


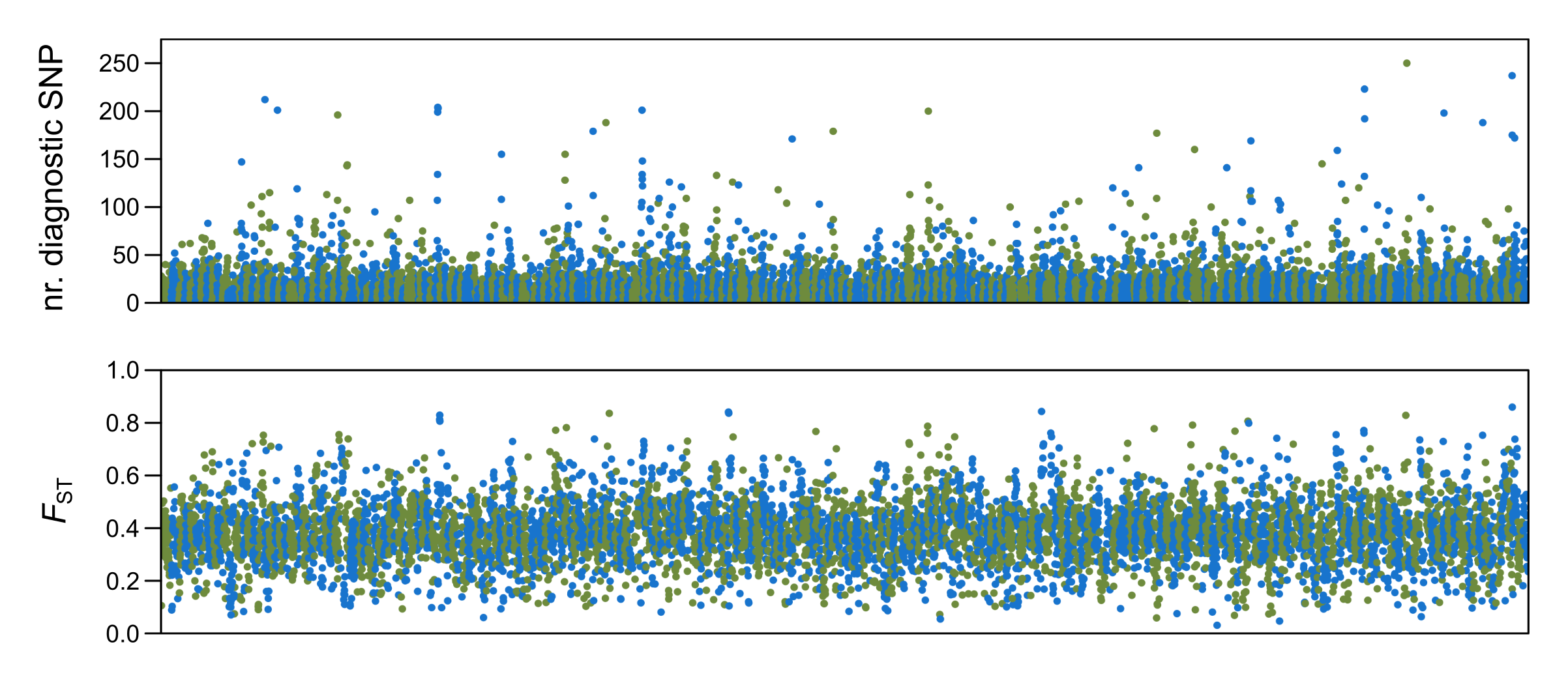
**Fig. S3.** Genetic differentiation between *P. bahiana* and *P. burmeisteri*, based on pool-sequencing data, for the largest 250 contigs in the reference assembly. On top, the distribution of counts of diagnostic alleles (ΔAF = 1.0). below the fixation index (*F*_ST_). Each dot corresponds to the estimate for an independent 10 kb window no overlap between consecutive windows). Alternating colours indicate alternating contigs.


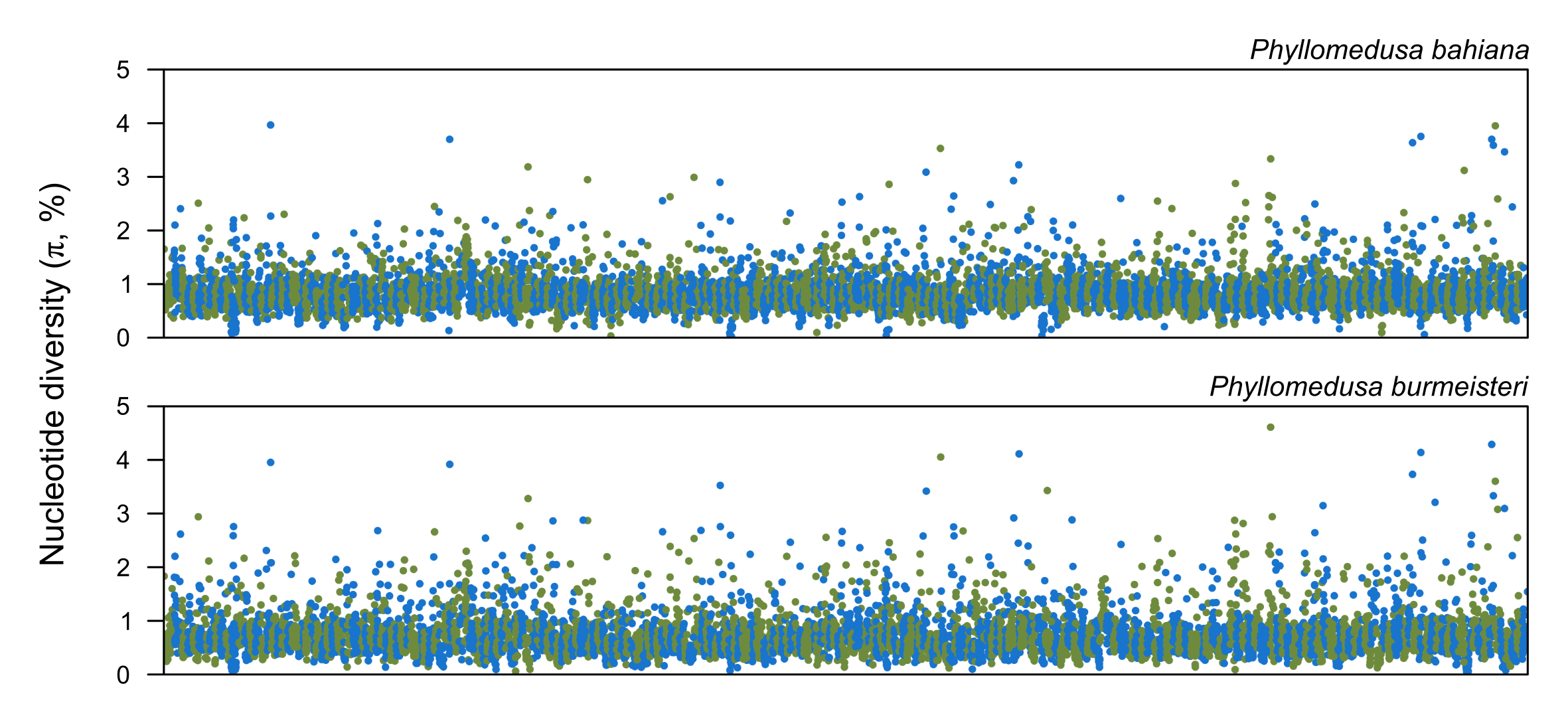
**Fig. S4.** Distribution of values of nucleotide diversity (π, %) for *P. bahiana* and *P. burmeisteri*, based on pool-sequencing data, for the largest 250 contigs in the reference assembly. Each dot corresponds to the estimate for an independent 10 kb window (no overlap between consecutive windows). Alternating colours indicate alternating contigs.


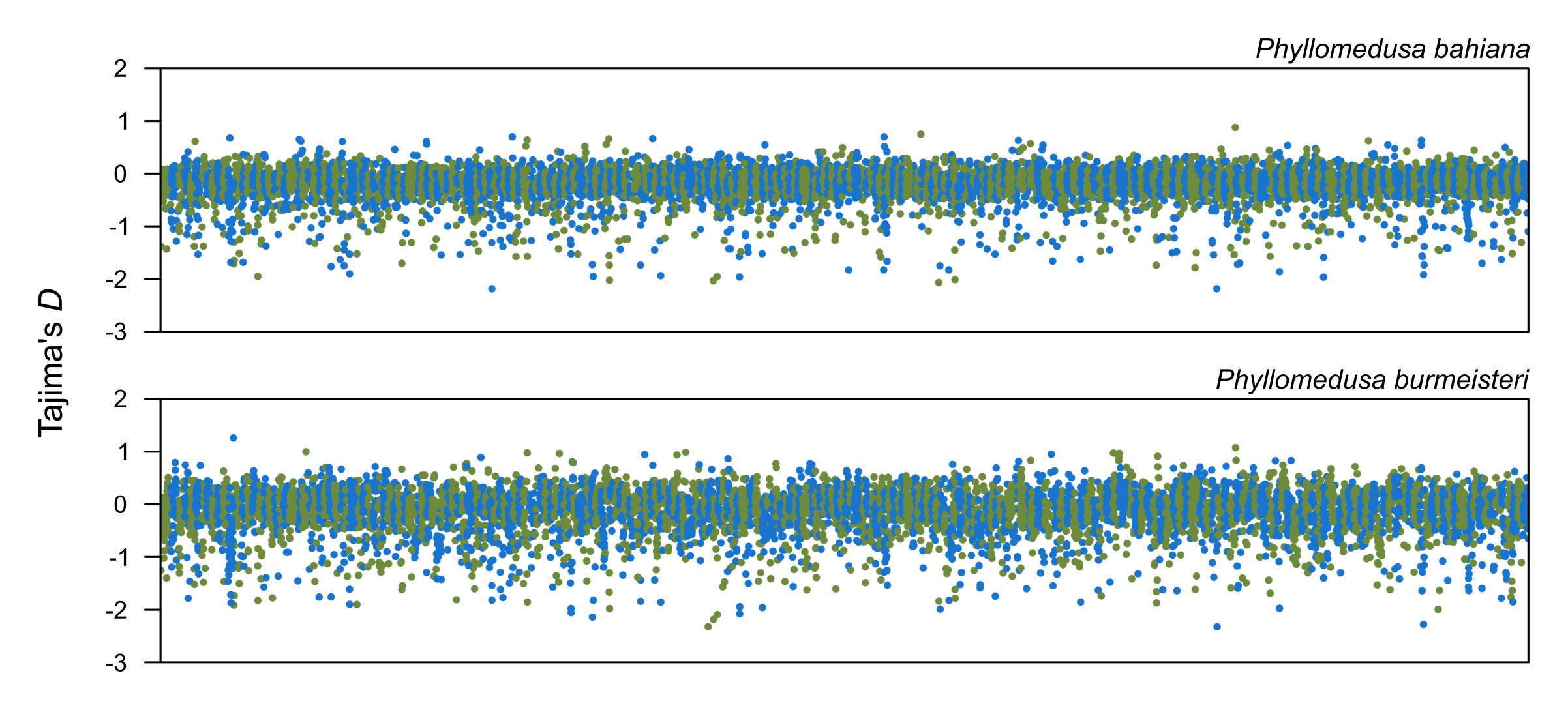
**Fig. S5.** Distribution of values of Tajima’s *D* for *P. bahiana* and *P. burmeisteri*, based on pool-sequencing data, for the largest 250 contigs in the reference assembly. Each dot corresponds to the estimate for an independent 10 kb window (no overlap between consecutive windows). Alternating colours indicate alternating contigs.

**Supplementary references**

Bredeson JV, Mudd AB, Medina-Ruiz S, Mitros T, Smith OK, Miller KE, Lyons JB, Batra SS, Park J, Berkoff KC, et al. Conserved chromatin and repetitive patterns reveal slow genome evolution in frogs. *BioRxiv*. 2021;10.1101/2021.10.18.464293

Ceschin DG, Pires NS, Mardirosian MN, Lascano CI, Venturino A. (2020). The *Rhinella arenarum* transcriptome: de novo assembly, annotation and gene prediction. Sci Rep. 2020;10(1):1-8.

Denton RD, Kudra RS, Malcom JW, Du Preez L, Malone JH. The African Bullfrog (*Pyxicephalus adspersus*) genome unites the two ancestral ingredients for making vertebrate sex chromosomes. BioRxiv. 2018;329847.

Edwards RJ, Tuipulotu DE, Amos TG, O'Meally D, Richardson MF, Russell TL, Vallinoto M, Carneiro M, Ferrand N, Wilkins MR, et al. Draft genome assembly of the invasive cane toad, *Rhinella marina*. Gigascience. 2018;7(9):giy095.

Furman BL, Cauret CM, Knytl M, Song XY, Premachandra T, Ofori-Boateng C, Jordan DC, Horb ME, Evans BJ. et al. A frog with three sex chromosomes that co-mingle together in nature: *Xenopus tropicalis* has a degenerate W and a Y that evolved from a Z chromosome. PLoS Genet. 2020;16(11):e1009121.

Gerchen JF, Reichert SJ, Röhr JT, Dieterich C, Kloas W, Stöck M. A single transcriptome of a green toad (*Bufo viridis*) yields candidate genes for sex determination and-differentiation and non-anonymous population genetic markers. PLoS One. 2016;11(5):e0156419.

Hammond SA, Warren RL, Vandervalk BP, Kucuk E, Khan H, Gibb EA, Pandoh P, Kirk H, Zhao Y, Jones M, et al. The North American bullfrog draft genome provides insight into hormonal regulation of long noncoding RNA. Nat Commun. 2017;8(1):1-8.

Huang L, Li J, Anboukaria H, Luo Z, Zhao M, Wu H. Comparative transcriptome analyses of seven anurans reveal functions and adaptations of amphibian skin. Sci Rep. 2016;6(1):1-11.

Katsura Y, Ikemura T, Kajitani R, Toyoda A, Itoh T, Ogata M, Miura I, Wada K, Wada Y, Satta, Y. Comparative genomics of *Glandirana rugosa* using unsupervised AI reveals a high CG frequency. Life Sci Alliance, 2021;4(5):e202000905.

Lamichhaney S, Catullo R, Keogh JS, Clulow S, Edwards SV, Ezaz T. A bird-like genome from a frog: Mechanisms of genome size reduction in the ornate burrowing frog, *Platyplectrum ornatum*. PNAS. 2021;118(11), e2011649118.

Li Y, Ren Y, Zhang D, Jiang H, Wang Z, Li X, Rao D. Chromosome-level assembly of the mustache toad genome using third-generation DNA sequencing and Hi-C analysis. Gigascience. 2019a;8(9):giz114.

Li J, Yu H, Wang W, Fu C, Zhang W, Han F, Wu H. Genomic and transcriptomic insights into molecular basis of sexually dimorphic nuptial spines in *Leptobrachium leishanense*. Nat Commun. 2019B;10(1):1-13.

Li Q, Guo Q, Zhou Y, Tan H, Bertozzi T, Zhu Y, Zhang, G. A draft genome assembly of the eastern banjo frog *Limnodynastes dumerilii dumerilii* (Anura: Limnodynastidae). Gigabyte. 2020;doi:10.46471/gigabyte.2

Liedtke HC, Garrido JG, Esteve-Codina A, Gut M, Alioto T, Gomez-Mestre I. De novo assembly and annotation of the larval transcriptome of two spadefoot toads widely divergent in developmental rate. G3: Genes, Genomes, Genet. 2019;9(8):2647-2655.

Liscano Martinez Y, Arenas Gómez CM, Smith JJ, Delgado JP. A Tree Frog (*Boana Pugnax*) Dataset of Skin Transcriptome for the Identification of Biomolecules with Potential Antimicrobial Activities. Data Br. 2020;32:106084.

Lu B, Jiang J, Wu H, Chen X, Song X, Liao W, Fu J. A large genome with chromosome‐scale assembly sheds light on the evolutionary success of a true toad (*Bufo gargarizans*). Molecular Ecol Res. 2021;21(4):1256-1273.

Ma WJ, Veltsos P, Sermier R, Parker DJ, Perrin N. Evolutionary and developmental dynamics of sex-biased gene expression in common frogs with proto-Y chromosomes. *Genome Biol*. 2018;*19*(1):1-17.

Montero-Mendieta S, Grabherr M, Lantz H, De la Riva I, Leonard JA, Webster MT, Vilà C. A practical guide to build de-novo assemblies for single tissues of non-model organisms: the example of a Neotropical frog. PeerJ. 2017;5,e3702.

Mudd AB. *Comparative genomics and chromosome evolution*. University of California, 2019, Berkeley.

Nürnberger B, Baird SJ, Čížková D, Bryjová A, Mudd AB, Blaxter ML, Szymura JM. A dense linkage map for a large repetitive genome: discovery of the sex-determining region in hybridizing fire-bellied toads (*Bombina bombina* and *Bombina variegata*). G3. 2021;11(12),jkab286.

Robertson LS, Cornman RS. Transcriptome resources for the frogs *Lithobates clamitans* and *Pseudacris regilla*, emphasizing antimicrobial peptides and conserved loci for phylogenetics. Mol Ecol Res*.* 2014;14(1):178-183.

Rodríguez A, Mundy NI, Ibáñez R, Pröhl H. Being red, blue and green: the genetic basis of coloration differences in the strawberry poison frog (*Oophaga pumilio*). BMC Genom. 2020;21(1):1-16.

Rogers RL, Zhou L, Chu C, Márquez R, Corl A, Linderoth T, Freeborn L, MacManes MD, Xiong Z, Zheng J, et al. Genomic takeover by transposable elements in the strawberry poison frog. Mol Biol Evol. 2018;35(12):2913-2927.

Seidl F, Levis NA, Schell R, Pfennig DW, Pfennig KS, Ehrenreich IM. Genome of *Spea multiplicata*, a rapidly developing, phenotypically plastic, and desert-adapted spadefoot toad. G3: Genes, Genomes, Genet. 2019;9(12):3909-3919.

Session AM, Uno Y, Kwon T, Chapman JA, Toyoda A, Takahashi S, Fukui A, Hitosaka A, Suzuki A, Kondo M, et al. Genome evolution in the allotetraploid frog *Xenopus laevis*. Nature. 2016;538(7625):336-343.

Streicher JW, Darwin Tree of Life Consortium. The genome sequence of the common toad, *Bufo bufo* (Linnaeus, 1758). Wellcome Open Res. 2021A;6(281):281.

Streicher JW, Darwin Tree of Life Consortium. The genome sequence of the common frog, *Rana temporaria* (Linnaeus 1758). Wellcome Open Res. 2021B;6(286):286.

Stuckert AM, Chouteau M, McClure M, LaPolice TM, Linderoth T, Nielsen R, Summers K, MacManes MD. The genomics of mimicry: gene expression throughout development provides insights into convergent and divergent phenotypes in a Müllerian mimicry system. Mol Ecol. 2021;30(16):4039-4061.

Sun YB, Xiong ZJ, Xiang XY, Liu SP, Zhou WW, Tu X-L, Zhong L, Wang L, Dong-Dong W, Bao-Lin Z, et al. Whole-genome sequence of the Tibetan frog *Nanorana parkeri* and the comparative evolution of tetrapod genomes. PNAS. 2015;112(11):E1257-E1262.

Vertebrate Genomes Project (2022, July, 5) *Dendropsophus ebraccatus*, hourglass treefrog, Version assembly_curated from 17 December 2021. GenomeArk. https://vgp.github.io/genomeark/Dendropsophus_ebraccatus.

Wu W, Gao YD, Jiang DC, Lei J, Ren JL, Liao WB, Deng C, Wang Z, Hillis DM, Zhang Y-P et al. Genomic adaptations for arboreal locomotion in Asian flying treefrogs. PNAS*.* 2022;119(13), e2116342119. doi:10.1073/pnas.2116342119
